# Electromagnetic forming of AA1060 sheet based on mixed forces generated by a three-coil dual-power system

**DOI:** 10.1038/s41598-023-49590-4

**Published:** 2024-03-02

**Authors:** Yanxin Li, Bo Tang, Yiliang Lv, Qi Xiong, Xiang Zhao

**Affiliations:** 1 College of Electrical Engineering & New Energy, China Three Gorges University, Yichang, 443002 China; 2 Hubei Provincial Engineering Technology Research Center for Power Transmission Line, China Three Gorges University, Yichang, 443002 China; 3 Wuhan National High Magnetic Field Center, Huazhong University of Science and Technology, Wuhan, 430074 China

**Keywords:** Electrical and electronic engineering, Mechanical engineering

## Abstract

The electromagnetic force used in electromagnetic forming is mainly divided into attraction and repulsion. Dual-coil attractive electromagnetic forming can be used in the field of sheet pit repair. However, the magnetic field and eddy current generated by the two coils compete with each other, and the energy utilization rate is low. Therefore, a compensation coil is introduced, and an electromagnetic forming scheme of a three-coil dual-power sheet based on mixed force is proposed and verified by simulation. It is found that the three-coil mixed force can effectively improve the competition between the magnetic field and eddy current. The loading of the mixing force is not a simple superposition of attraction and repulsion, but the mutual promotion of the two. The forming displacement of the three-coil mixed force forming scheme is 582% higher than that of the dual-coil attraction forming scheme, and 89% higher than that of the attract first and then repel forming scheme. The forming effect of the three-coil mixing force is related to the number of turns of the compensation coil. The research results can improve the energy utilization rate of electromagnetic forming and provide a new idea for the loading scheme of electromagnetic forming force field.

## Introduction

Electromagnetic forming (EMF) is a high-energy, high-speed forming technology^1–3^, which realizes the forming and processing of materials through electromagnetic force^4,5^. Compared with the traditional method, it can effectively improve the forming limit of the workpiece^6,7^.

The loading of electromagnetic force in the electromagnetic forming process is very flexible, and the combination of coil and current can produce repulsion force or attraction in the workpiece. In the single pulse-single coil electromagnetic forming system, the repulsive force is far greater than the attraction^8,9^. In repulsive forming, the coil needs to bear large stress and is easy to break. Therefore, Deng et al. proposed that the pulse current with a slow rising edge and accelerating falling edge can generate attractive electromagnetic force in the workpiece^10^. Cao et al. and Xiong et al.^11,12^ proposed the use of a dual-frequency current method for attractive electromagnetic forming. This method greatly improves the flexibility of electromagnetic force loading in electromagnetic forming.

The dual-frequency current method can effectively generate attractive forming in a single coil. Xiong et al. achieved tube bulging and flaring^13,14^. However, the circuit parameters of the single coil are fixed, which requires extremely high discharge parameters. If the two sets of power supply parameters do not match slightly, it is difficult to form the current required for attraction. On this basis, Ouyang et al.^15^ and Xiong et al.^16–18^ successively proposed the use of a dual-coil dual-power system to improve this problem and successfully applied it to tube and sheet processing. Compared with the single coil system, the double coil system is more flexible in the loading of the electromagnetic force, but there are also many problems. Xiong et al. proposed that there is eddy current competition in the attractive forming of double coil sheets and studied its mechanism^19^.

In view of the above problems, this paper proposes a three-coil dual-power sheet forming system. Under the same power supply parameters, compared with the dual-coil attraction forming scheme and the attract first and then repel forming scheme. Its advantage is that it can effectively improve the eddy current competition and magnetic field competition in the attractive electromagnetic forming process. Thereby improving energy utilization, improving the stress state on the coil skeleton.

## Methods

In this paper, the solenoid coil is selected as the driving coil. To simplify the calculation, the solenoid coil can be equivalent to a finite number of closed ring stacking^20^. From the nature of the electromagnetic field, and the geometric relationship between the closed ring and the cylindrical sheet, it can be seen that the electromagnetic field load source and structure of the driving coil and the workpiece are axisymmetric, which can be transformed into a two-dimensional axisymmetric model. From Maxwell's equations of electromagnetic induction equation,

1 $$ \nabla \times {\varvec{E}}_{\phi } = - \frac{{\partial {\varvec{B}}}}{\partial t} + \nabla \times \left( {{\varvec{v}} \times {\varvec{B}}} \right) $$

${\varvec{E}}_{\phi }$ is the circumferential electric field intensity, $\nabla \times {\varvec{E}}_{\phi }$ is the curl of the circumferential electric field intensity, $- \partial {\varvec{B}}/\partial t$ is the induced electromotive force, ${\varvec{v}}$ is the workpiece speed, and $\nabla \times \left( {{\varvec{v}} \times {\varvec{B}}} \right)$ is the dynamic electromotive force.

2 $$ {\varvec{J}}_{\phi } = \gamma {\varvec{E}}_{\phi } $$

${\varvec{J}}_{\phi }$ is the circumferential current density, and $\gamma$ is the conductivity of the sheet. Equations (1) and (2) show the cause of induced eddy current in the sheet during electromagnetic forming.

The electromagnetic force is generated by the interaction between the induced eddy current in the sheet and the space magnetic field. According to the expression of the electromagnetic force.

3 $$ {\varvec{f}} = {\varvec{J}} \times {\varvec{B}} $$

${\varvec{J}}$ is the eddy current density in the sheet, ${\varvec{B}}$ is the magnetic field, and ${\varvec{f}}$ is the density of the electromagnetic force at a point in the sheet. The electromagnetic force is divided into two parts, radial and axial.

4 $$ {\varvec{f}}_{r} = {\varvec{J}}_{\phi } \times {\varvec{B}}_{z} $$

5 $$ {\varvec{f}}_{z} = {\varvec{J}}_{\phi } \times {\varvec{B}}_{r} $$

${\varvec{f}}_{r}$ is the radial electromagnetic force density of the sheet, and ${\varvec{f}}_{z}$ is the axial electromagnetic force density of the sheet. The sheet metal forming process is mainly affected by the axial electromagnetic force.

**Figure 1:**
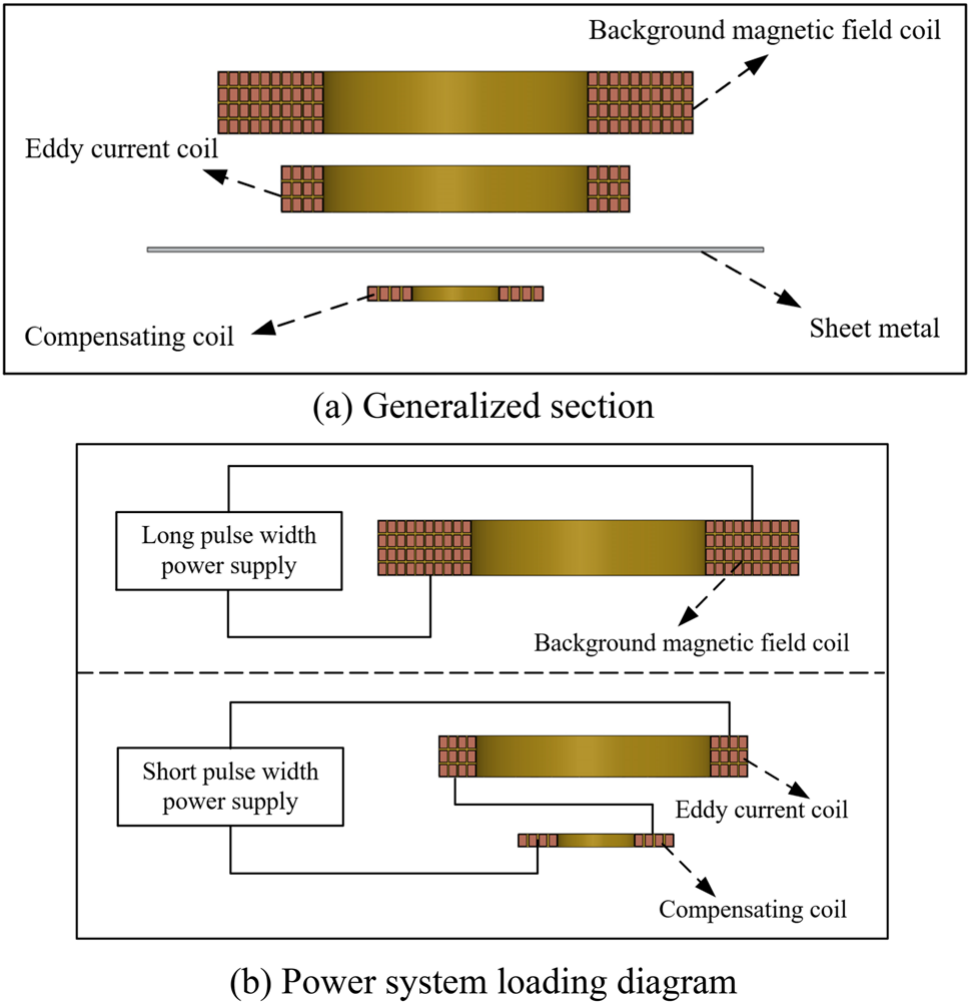
The three-coil dual-power sheet forming system.

**Figure 2:**
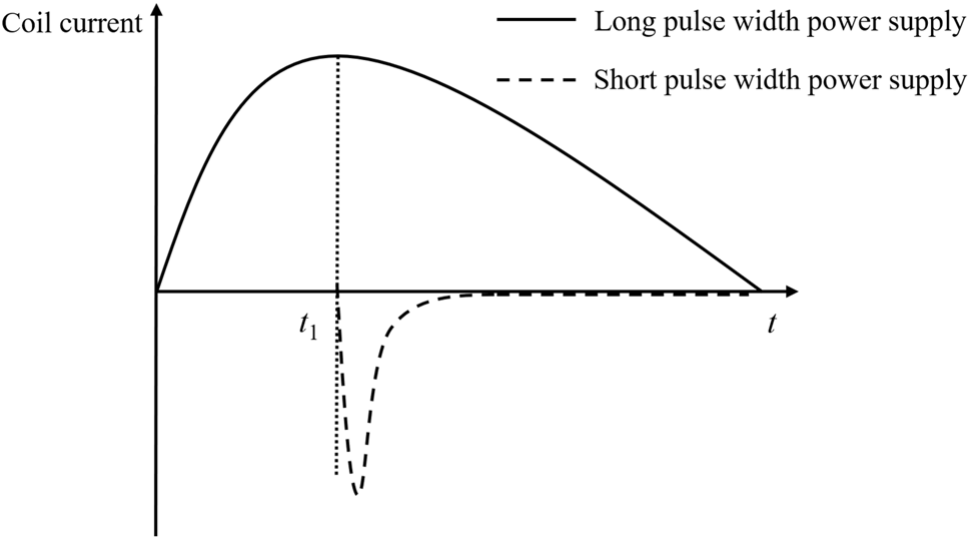
Current timing loading diagram.

The schematic diagram of the electromagnetic forming system of the three-coil dual power supply and the power supply loading diagram are shown in Fig. 1. The current loading sequence diagram is shown in Fig. 2.

When $t < t_{1}$,the long pulse width current is loaded into the background magnetic field coil to generate the background magnetic field. When the current reaches the peak (i.e., $t_{1}$), the short pulse width current is loaded into the eddy current coil and the compensation coil. The induced eddy current generated by the background magnetic field coil in the workpiece is opposite to the induced eddy current generated by the eddy current coil in the workpiece, and the eddy current competition occurs at this time. When the short pulse current is loaded into the coil, the short pulse current ***I***_s_ interacts with the radial background magnetic field ***B***_r_, which will produce a huge axial Electromagnetic force and may destroy the coil skeleton structure. The influence of magnetic field competition, eddy current competition and coil tooling is unfavorable to sheet forming, so the compensation coil is introduced to improve the magnetic field competition and eddy current competition.

The direction of the background magnetic field is defined as the positive direction of the magnetic field, and the direction of the induced eddy current generated by the eddy current coil is defined as the positive direction of the induced eddy current, so the axial electromagnetic force on the sheet is,

6 $$ {\varvec{f}}_{z} = \left\{ {\begin{array}{*{20}l} { - {\varvec{J}}_{\phi L} \times {\varvec{B}}_{rL} ,} \hfill & {\quad t \le t_{1} } \hfill \\ {\left( { - {\varvec{J}}_{\phi L} + {\varvec{J}}_{\phi S} + {\varvec{J}}_{\phi O} } \right) \times \left( {{\varvec{B}}_{rL} - {\varvec{B}}_{rS} + {\varvec{B}}_{rO} } \right),} \hfill & {\quad t > t_{1} } \hfill \\ \end{array} } \right. $$

${\varvec{J}}_{\phi L}$ is the induced eddy current generated by the background magnetic field coil in the workpiece, ${\varvec{B}}_{rL}$ is the magnetic field generated by the background magnetic field coil, ${\varvec{J}}_{\phi S}$ is the induced eddy current generated by the eddy current coil in the sheet, and ${\varvec{B}}_{rS}$ is the magnetic field generated by the eddy current coil. ${\varvec{J}}_{\phi S}$ is the induced eddy current generated by the compensation coil in the sheet, and ${\varvec{B}}_{rO}$ is the magnetic field generated by the compensation coil.

1. Improve the attractive forming magnetic field competition

**Figure 3:**
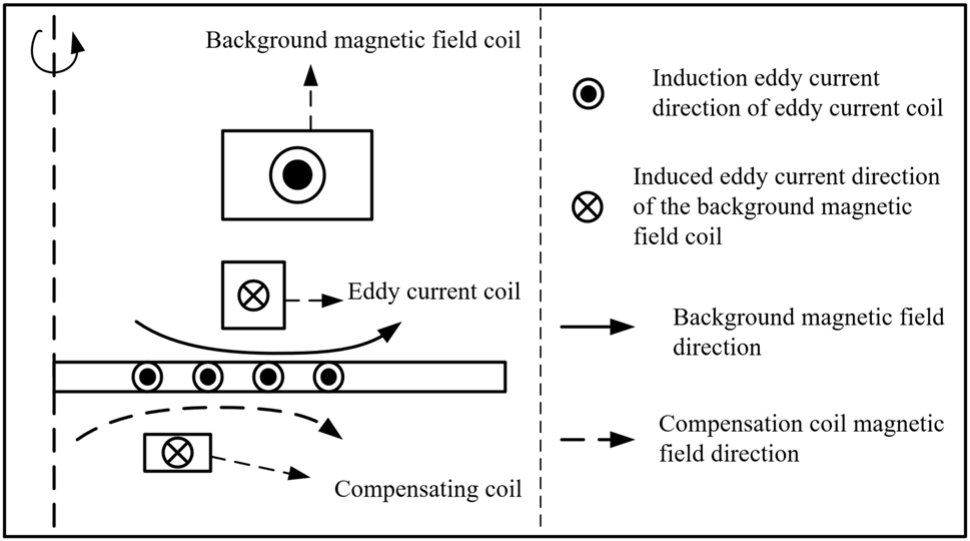
The three-coil decoupling electromagnetic environment.

To facilitate the analysis of the magnetic field and eddy current environment in sheet metal forming, the three coils are decoupled here. Figure 3 shows the magnetic field and eddy current decoupling the electromagnetic environment of the forming system after introducing the compensation coil. The magnetic field required for sheet forming is provided by the background magnetic field coil. The direction of the magnetic field generated by the compensation coil is consistent with the direction of the background magnetic field. The double-coil electromagnetic attraction system requires the interaction between the magnetic field in the positive direction and the induced eddy current in the positive direction. The eddy current coil will produce a magnetic field in the opposite direction, which will affect the magnetic field environment in the attractive forming process of the sheet. Therefore, the magnetic field competition relationship in the process of double coil attractive forming is $\left( {{\varvec{B}}_{rL} - {\varvec{B}}_{rS} } \right)$. The compensation coil generates a magnetic field in the positive direction, which is exactly the magnetic field required for sheet forming. Therefore, the introduction of a compensation coil can improve the magnetic field competition, thereby improving the magnetic field environment during the workpiece forming process. After the compensation coil is introduced, the magnetic field competition relationship is $\left( {{\varvec{B}}_{rL} - {\varvec{B}}_{rS} + {\varvec{B}}_{rO} } \right)$.

(2) Improve the attractive forming vortex competition

In the dual-coil attractive system, the sheet forming needs the induced eddy current in the positive direction. The background magnetic field coil will generate an induced eddy current in the opposite direction in the sheet. Therefore, there is a competitive relationship between the induced eddy current generated by the background magnetic field coil and the induced eddy current generated by the eddy current coil, and the competitive relationship is $\left( { - {\varvec{J}}_{\phi L} + {\varvec{J}}_{\phi S} } \right)$. The compensation coil generates an induced eddy current in the positive direction in the sheet, which is exactly the eddy current required for sheet forming. Therefore, the introduction of the compensation coil can improve the eddy current competition, and the eddy current competition relationship after the introduction of the compensation coil is $\left( { - {\varvec{J}}_{\phi L} + {\varvec{J}}_{\phi S} + {\varvec{J}}_{\phi O} } \right)$.

(3) Optimize the coil stress environment

Since the current is directly introduced into the coil, the coil is subjected to large stress when the power supply parameters are too large, and it is easy to break or even explode. In the three-coil mixed force system, the eddy current coil and the compensation coil are in series operation, which can realize the voltage division. At this time, the current in the compensation coil will be greatly reduced, thereby improving its stress environment.

## The three-coil dual-power supply forming simulation model

### Power system design

**Figure 4:**
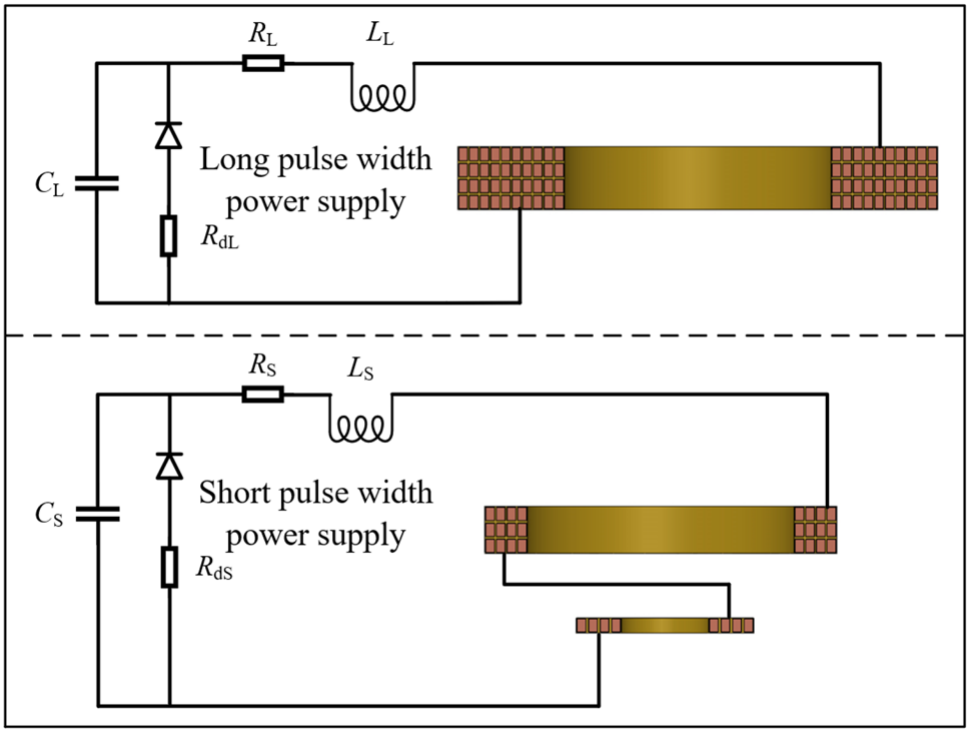
Power system topology diagram.

Figure 4 is the power system topology. The long and short pulse width power supply systems are all RLC circuits, which are energized to the three coils in combination with the crowbar circuit. The discharge parameters are shown in Table 1.

**Table 1:** Power system discharge parameters.

Electrical discharge parameters	Long pulse width power supply		Short pulse width power supply	
	Parameter	Value	Parameter	Value
Discharge voltage	*U*_L_/kV	0–25	*U*_S_/kV	0–25
Capacitance	*C*_L_/μF	2880	*C*_S_/μF	160
Line resistance	*R*_L_/mΩ	130	*R*_S_/mΩ	15
Line inductance	*L*_L_/μH	415	*L*_S_/μH	7
Crowbar resistance	*R*_dL_/mΩ	260	*R*_dS_/mΩ	50–200

### Coil design

**Figure 5:**
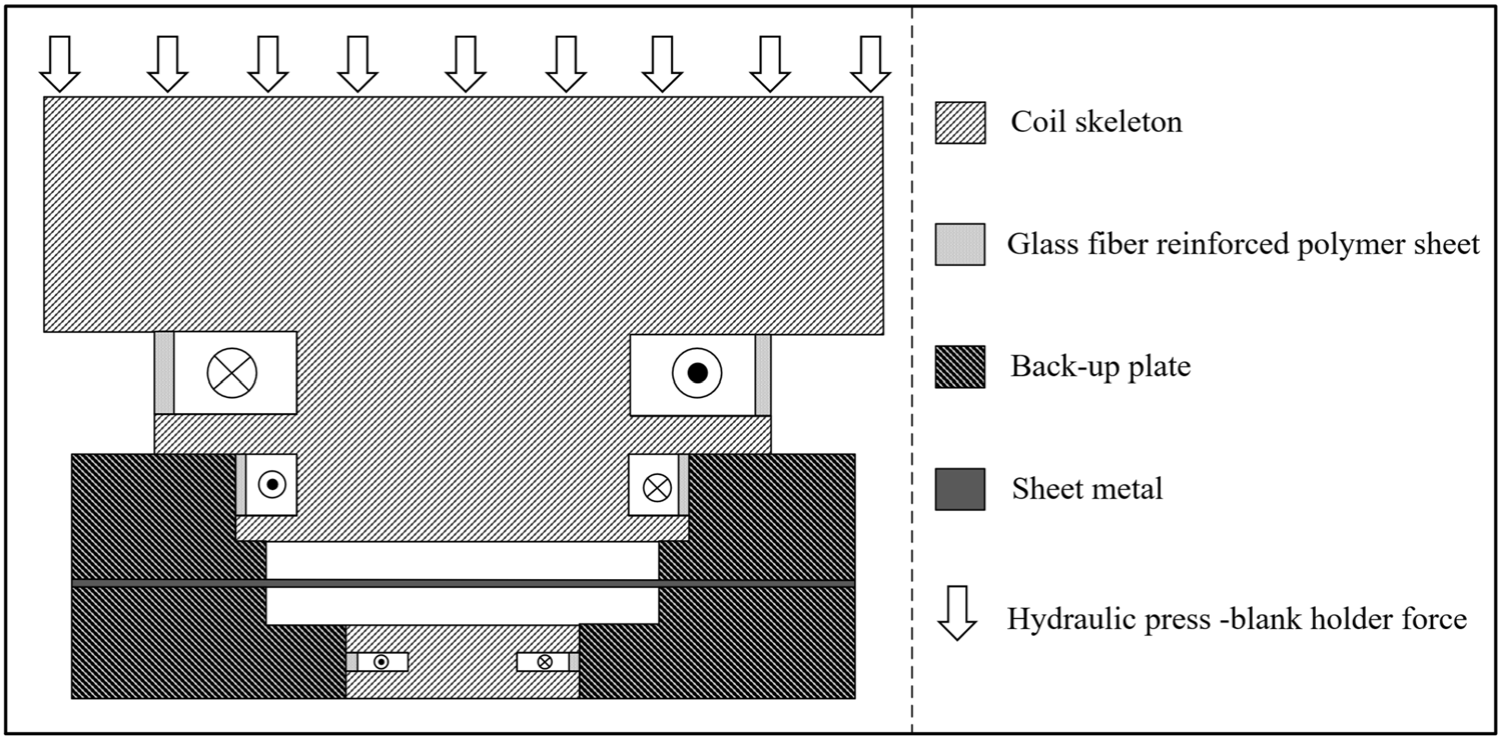
System overall scheme.

According to the above coil parameter design, the overall scheme of the system is shown in Fig. 5.

**Figure 6:**
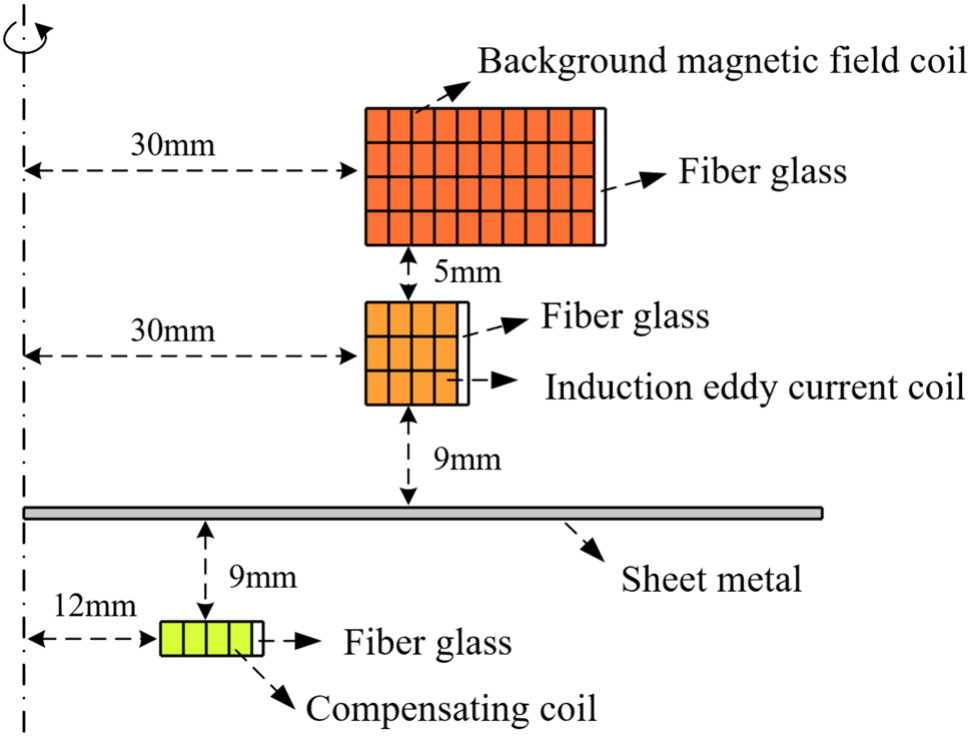
The coil size.

In this system, the coil is fixed by the skeleton, and the hydraulic press acts on the coil skeleton to make the whole fixed in the axial direction. The coil will generate stress during the energization process, the coil will move in the radial direction, so the glass fiber is used to fix the coil in the radial direction. The specific dimensions and parameters of the coil and the workpiece are shown in Fig. 6 and Table 2.

**Table 2:** Parameters of coils and workpiece.

Module	Parameter	Value
Background magnetic field coil (copper)	Inner diameter	30 mm
	Number of turns	4 × 10 = 40
	Single-turn cross-sectional area	2 mm × 3 mm = 6 mm^2^
Eddy current coil (copper)	Inner diameter	30 mm
	Number of turns	3 × 4 = 12
	Single-turn cross-sectional area	2 mm × 3 mm = 6 mm^2^
Compensating coil (copper)	Inner diameter	12 mm
	Number of turns	1 × 4 = 4
	Single-turn cross-sectional area	2 mm × 3 mm = 6 mm^2^
Material properties (copper)	Density	8960 kg/m^3^
	Conductivity	5.998 × 10^7^ S/m
Sheet metal (AA1060-O)	Radius	70 mm
	Thickness	1 mm
Material properties (AA1060-O)	Density	2710 kg/m^3^
	Conductivity	3.72 × 10^7^ S/m
	Young's modulus	69 GPa
	Poisson's ratio	0.33

In the process of electromagnetic forming, and the stress–strain relationship has a great influence on the deformation of the workpiece. Therefore, the Cowper-Symonds constitutive model is used to analyze the deformation of the sheet^21^.

7 $$ \sigma = \sigma_{y} \left[ {1 + \left( {\frac{\varepsilon }{C}} \right)^{m} } \right] $$

where $\sigma_{y}$ and $\varepsilon$ are quasi-static stress and plastic strain rate. For aluminum alloy sheet, $C = 6500s^{ - 1}$, m = 0.25.

### The algorithm flow of the model

**Figure 7:**
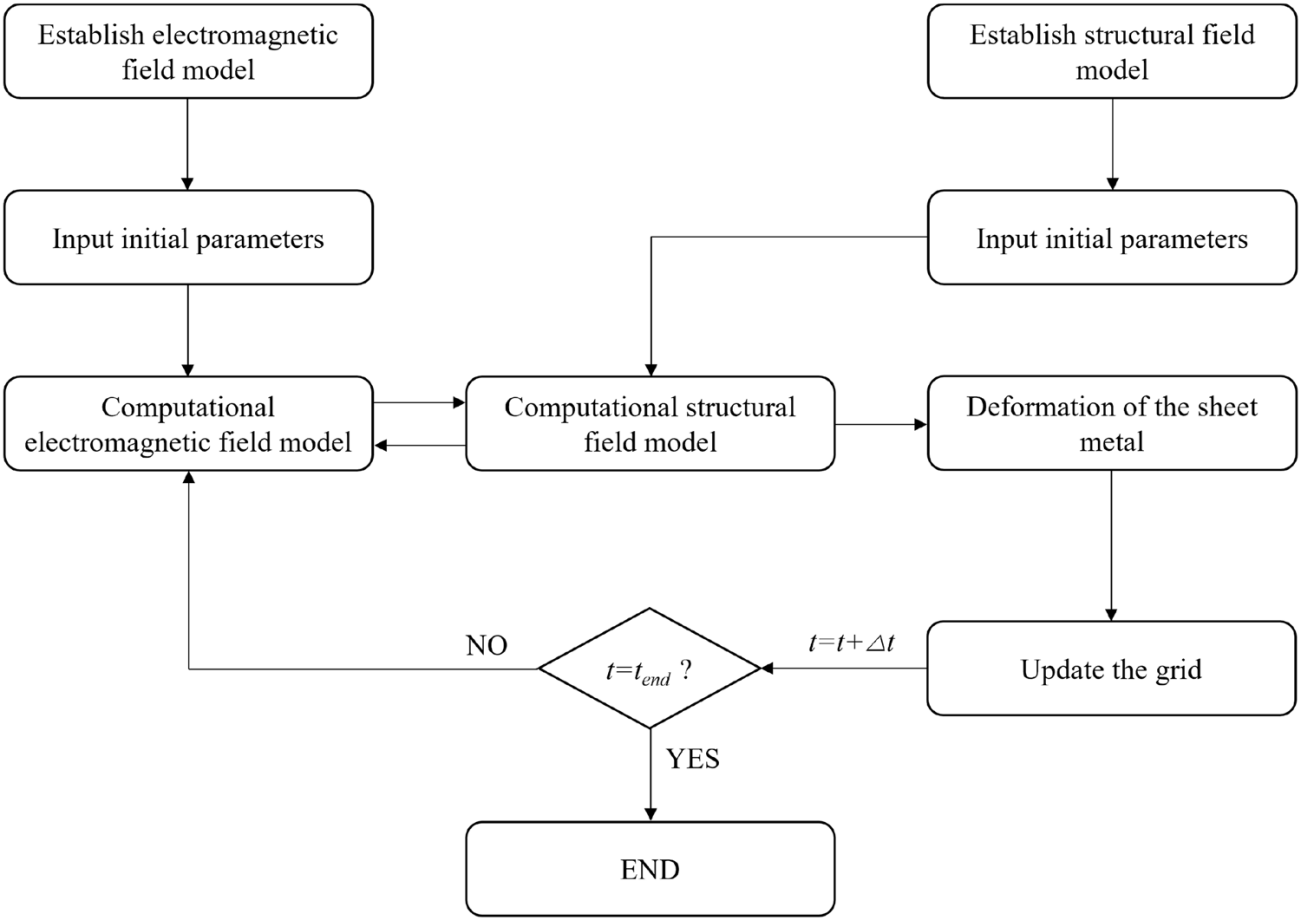
Sheet metal forming algorithm flow.

At present, the finite element method is the most effective simulation tool for electromagnetic forming. In this paper, a fully coupled model of electromagnetic forming is established^22^, and the algorithm flow of the model is shown in Fig. 7.

The complete solution process in a time step is to solve the current and voltage in the coil through the global equation. Secondly, the magnetic field distribution, eddy current and the electromagnetic force of the solution domain are calculated according to the electromagnetic field model. The electromagnetic force is loaded onto the workpiece in the form of body load in the structural field model. The workpiece deformation is calculated according to the material strain model. Finally, the displacement information of the workpiece is transmitted to the mobile network module to update the model.

## Results and analysis

**Figure 8:**
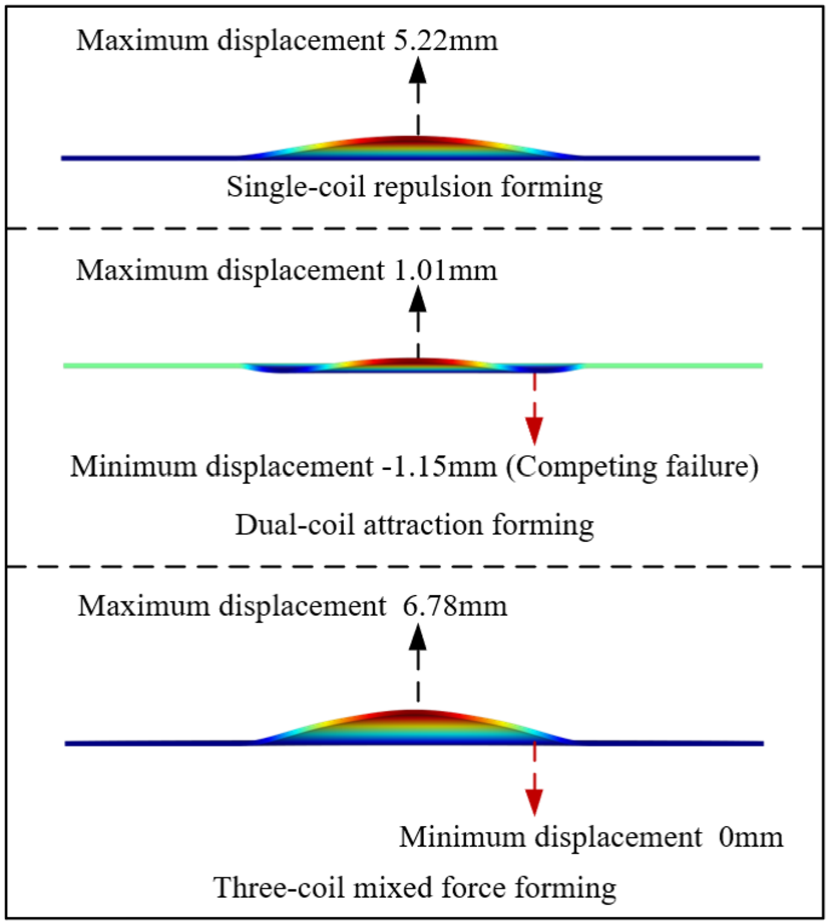
Comparison of forming effects.

The voltage combination used in the system is long pulse width voltage U_L_ = 6.15 kV, short pulse width voltage U_S_ = 6.8 kV, and crowbar resistance R_dL_ = 50 mΩ. Figure 8 is the comparison of the forming effects of the three forming schemes. The maximum displacement of the single-coil repulsion forming is 5.22 mm, the dual-coil attraction forming is 1.01 mm, and the three-coil mixed force forming is 6.89 mm. The forming displacement is 582% higher than that of the dual-coil attraction forming. The concave area generated during forming is defined as the competitive failure area. Due to the poor magnetic field competition and eddy current competition during the forming of the dual-coil attraction, the forming force field is not good, resulting in a competitive failure area. The three-coil mixing force forming successfully eliminates the competitive failure area. It can greatly improve the forming force field of the dual-coil attraction forming, thereby improving the forming effect.

### Magnetic field competition and eddy current competition

The axial electromagnetic force required for sheet forming is generated by the radial magnetic field and the induced eddy current in the workpiece. The radial magnetic field generated by the background magnetic field coil is opposite to the radial magnetic field generated by the eddy current coil. Therefore, the radial magnetic field has a competitive relationship in the dual-coil system. When the long pulse width current is on, the induced eddy current generated in the sheet is opposite to the direction of the induced eddy current required for forming, which is unfavorable for sheet forming. At this time, eddy current competition will occur.

**Figure 9:**
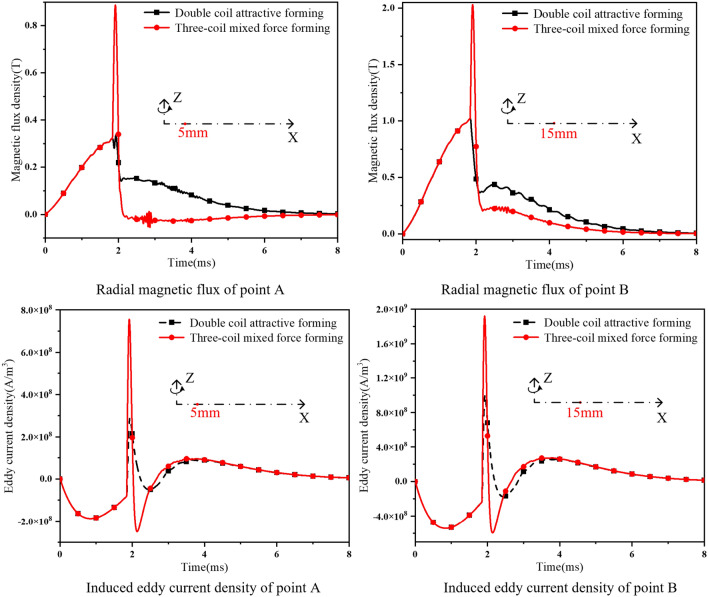
Comparison of radial magnetic flux density and eddy current density.

This paper introduces a compensation coil to improve this problem. Taking the center point of the sheet as the origin, point A (5 mm from the center of the sheet) and point B (15 mm from the center of the sheet) are selected for research. The radial magnetic flux and induced eddy current of point A and point B in the forming process of the two forming methods are shown in Fig. 9 and Table 3 respectively.

**Table 3:** Data comparison of point A and point B.

Forming method	Peak magnetic flux density (T)		Peak eddy current density (A/m^2^)	
	Point A	Point B	Point A	Point B
Three coil mixing force	0.89	2.03	7.31 × 10^8^	2.16 × 10^9^
Double coil attraction	0.32	1.02	3.05 × 10^8^	1.13 × 10^9^
Lifting capacity	178%	99%	139%	91%

**Figure 10:**
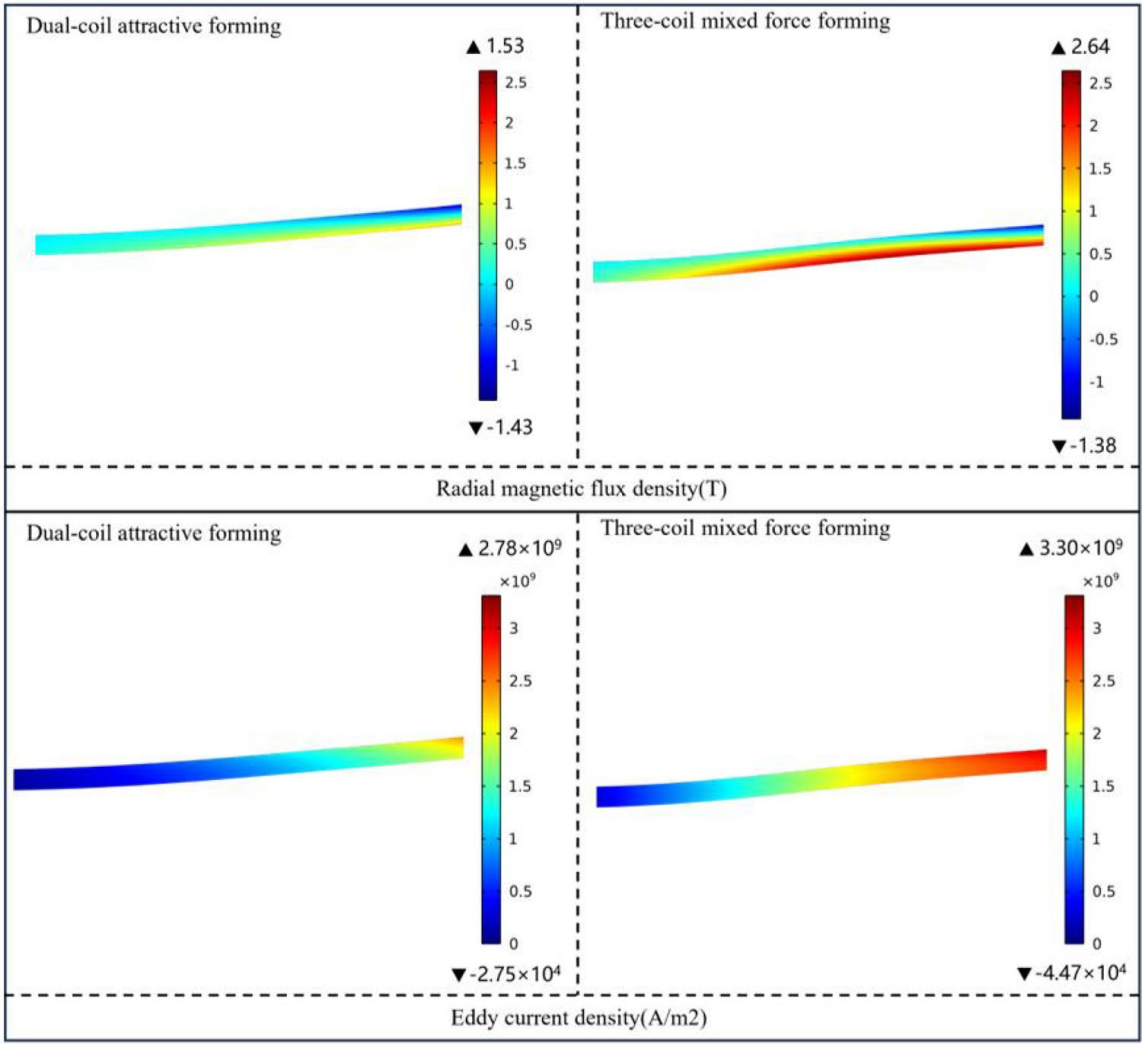
The cloud diagram of magnetic induction intensity and current density.

Compared with the double-coil attractive forming, the magnetic field in the three-coil mixed force scheme is increased by 178%, and the eddy current is increased by 91%. By comparing the cloud diagram of radial magnetic induction intensity and current density in the stress concentration area of sheet forming. As shown in Fig. 10. It can be seen that the introduction of the compensation coil can effectively improve the magnetic field competition and eddy current competition in the scheme proposed in this paper.

**Figure 11:**
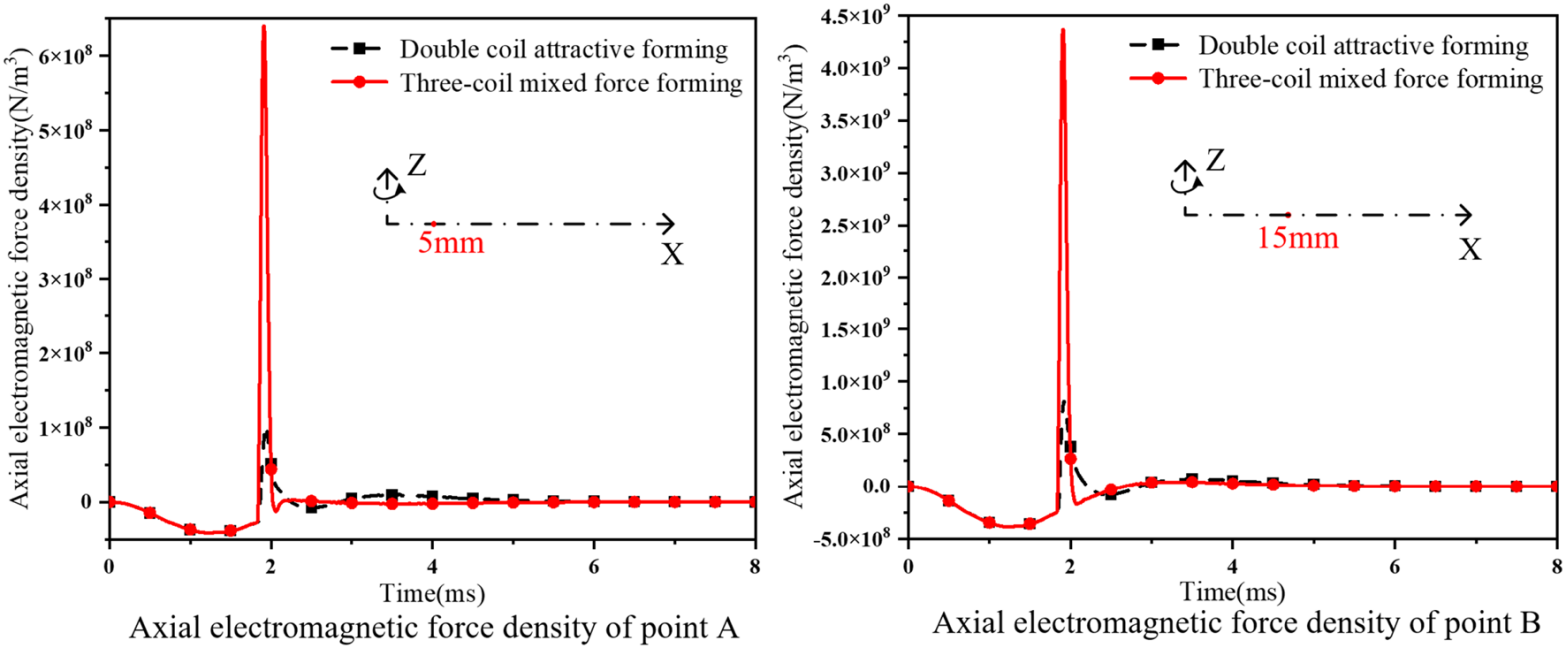
Comparison of axial electromagnetic force density.

It can be seen from Formula (5) that the magnetic field and eddy current increase, and the electromagnetic force will increase accordingly. The axial electromagnetic force density of points A and B in the forming process of the two forming methods varies with time, as shown in Fig. 11. at point A, the peak electromagnetic force density during the dual-coil attractive forming process is 1.02 × 10^8^ N/m^3^, and the peak electromagnetic force density during the three-coil mixed force process is 6.45 × 10^8^ N/m^3^, which is increased by 502%. At point B, the peak electromagnetic force density in the dual-coil attractive forming process is 8.18 × 10^8^ N/m^3^, and the peak electromagnetic force density in the three-coil mixed force process is 4.39 × 10^9^ N/m^3^, which is increased by 436%.

**Figure 12:**
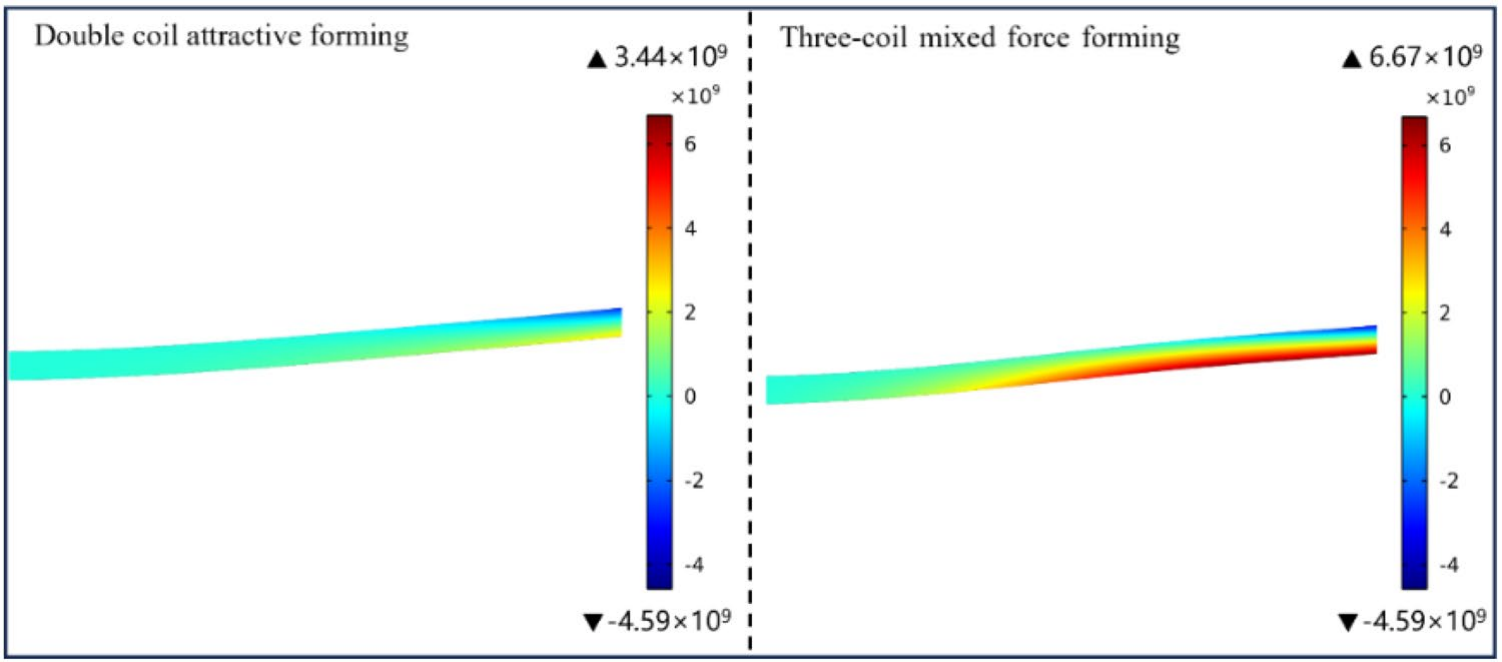
The electromagnetic force density cloud diagram at the maximum time.

The axial electromagnetic force density cloud diagram at the maximum moment is shown in Fig. 12. The three-coil mixed forming scheme increases the axial electromagnetic force density from the original 3.44 × 10^9^ N/m^3^ to 6.67 × 10^9^ N/m^3^. The three-coil mixed forming scheme proposed in this paper can effectively improve the axial electromagnetic force.

If the energy used in the two forming methods is the same, the three-coil mixed force forming can greatly improve the energy utilization efficiency in the electromagnetic forming process.

### Comparison of forming schemes and influence of coil turns on forming

**Figure 13:**
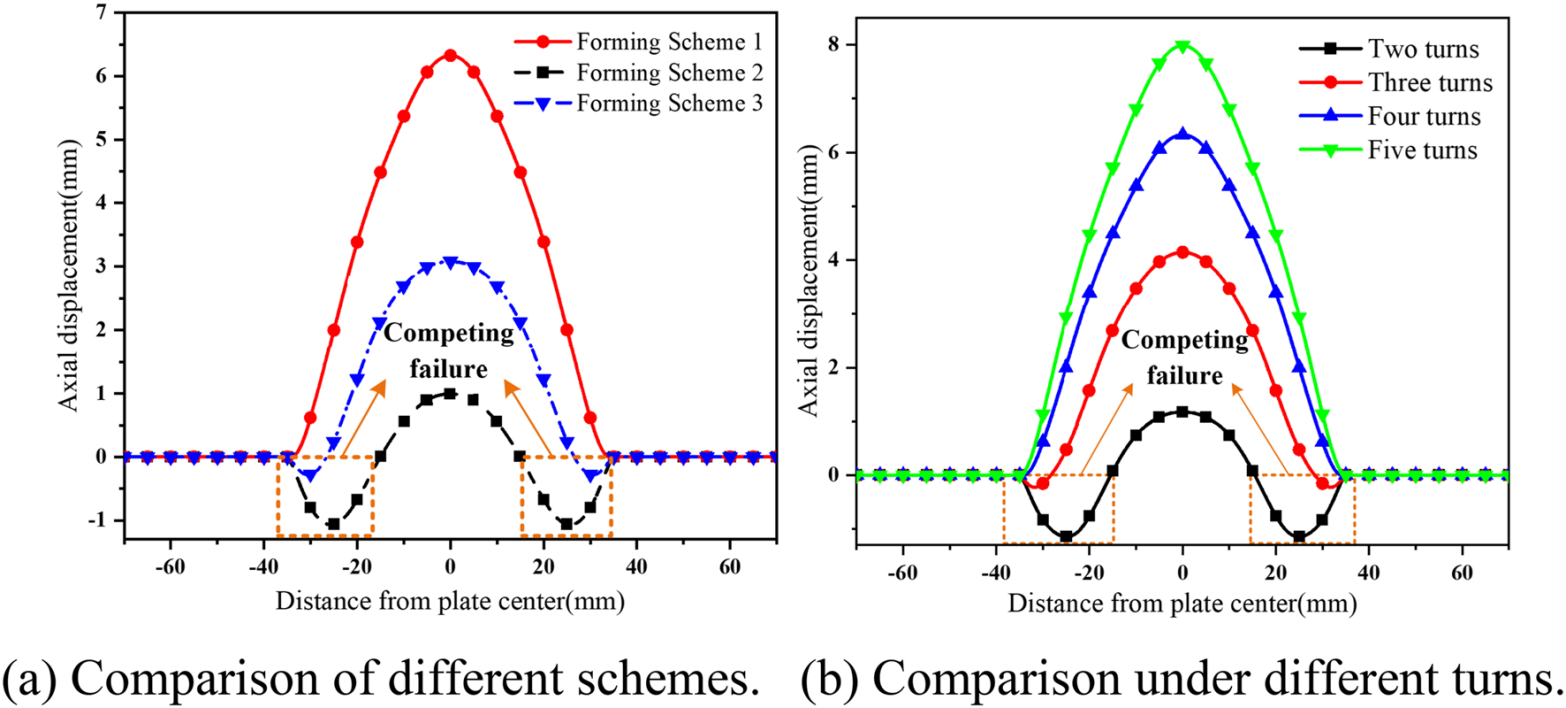
Comparison of axial displacement.

The loading of electromagnetic force in the mixed force forming process introduced in this paper is ${\varvec{f}}_{z} = \left( { - {\varvec{J}}_{\phi L} + {\varvec{J}}_{\phi S} + {\varvec{J}}_{\phi O} } \right) \times \left( {{\varvec{B}}_{rL} - {\varvec{B}}_{rS} + {\varvec{B}}_{rO} } \right)$, which is the increase of force caused by the improvement of the electromagnetic environment in the forming process. The process is different from the simple superposition of attraction and repulsion. In this section, the final forming effect of the three-coil mixed force forming (Forming Scheme 1), the dual-coil attraction forming (Forming Scheme 2), the attract first and then repel forming (Forming Scheme 3) is compared. The comparison results are shown in Fig. 13a.

As shown in Fig. 13a, the forming displacement of Scheme 1 (6.89 mm) is 582% higher than that of Scheme 2 (1.01 mm) and 89% higher than that of Scheme 3 (3.64 mm). There is a concave area caused by a competitive failure in Scheme 2 and Scheme 3, and there is no competitive failure area in Scheme 1.

The magnetic field and induced eddy current of dual-coil attraction forming are improved after the introduction of the compensation coil, so as to improve the force field in the forming process and improve the forming effect. The discharge parameters of Scheme 1 and Scheme 2 are the same, and the discharge energy is the same, *W*_1_ = *W*_2_ = *W*_L_ + *W*_S_ = 1/2*C*_L_*U*_L_^2^ + 1/2*C*_S_*U*_S_^2^ = 58,163.6 J. The discharge energy of Scheme 3 is *W*_3_ = *W*_L_ + *W*_S_ + *W*_S_ = 61,862.8J. The discharge energy (*W*_3_ > *W*_2_ = *W*_1_) and forming displacement (Scheme 1 > Scheme 3 > Scheme 2) prove that the three-coil mixed force forming scheme can improve the energy utilization rate.

As shown in Fig. 13b, When the number of turns of the compensation coil is less than 3, the sheet-forming effect of the three-coil dual-power mixed force-forming scheme is poor, and there is still a competitive failure area. In the process of increasing the number of turns, when the number of turns is greater than 3, the competitive failure area disappears and the displacement becomes larger and larger. Therefore, the larger the number of turns of the compensation coil, the better the forming effect of the sheet.

## Coil stress calibration

When the coil is working, the huge pulse current will produce a strong electromagnetic force inside the coil, which may cause the coil to rupture or even explode. Therefore, the coil must be checked for stress. In the three-coil mixed force forming, the coils will have coupling effects with each other. After analysis, this effect is positive. On the one hand, the introduced compensation coil and the eddy current coil are in series, sharing part of the electromagnetic energy, so the stress environment of the eddy current coil is improved. On the other hand, for single-coil repulsion forming, to increase the forming amount, the electric discharge voltage must be increased. While the discharge voltage is increased, the magnetic stress borne by the magnet is also increased in a square relationship. The three-coil mixed force forming scheme does not increase the discharge voltage while increasing the forming amount, so the stress level borne by the coil will not rise sharply.

The stress of the coil is simulated utilizing the finite element model. In the case of the same power supply parameters, the stress of three-coil mixed force forming, dual-coil attractive forming and single-coil repulsion forming is compared when the workpiece is running. The basic principle and simulation calculation method of coil stress verification is detailed in the reference^23^.

### Background magnetic field coil and eddy current coil

**Figure 14:**
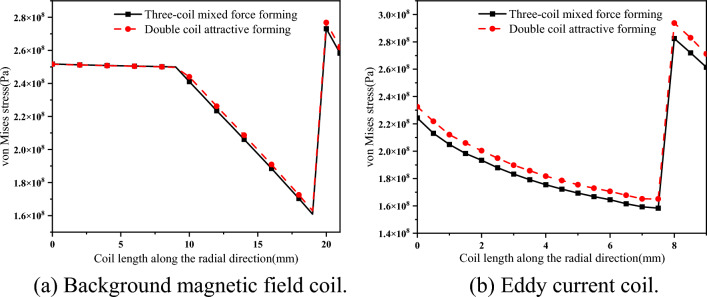
Comparison of coil stress under different schemes.

When the background magnetic field reaches the peak value, as shown in Fig. 14a, the stress distribution in the cross-section direction of the background magnetic field coil in two cases is calculated. When the short pulse width current reaches the peak value, the coil stress is the largest. As shown in Fig. 14b, the stress distribution in the cross-sectional direction of the eddy current coil is calculated in two cases.

From Fig. 14a, the stress difference between the background magnetic field coil in the three-coil mixed force forming and the dual-coil attraction forming is not large, and the three-coil mixed force forming scheme is slightly smaller. The circuit parameters of the two forming schemes are the same, so the overall electromagnetic environment of the coil is not much different. However, in the three-coil mixed force forming, due to the partial pressure of the compensation coil and the eddy current coil, the current of the eddy current coil will be reduced, and the induced eddy current generated in the background magnetic field coil caused by the current change in the eddy current coil will also be reduced. Therefore, the three-coil mixed force forming scheme has a certain optimization effect on the stress environment of the background magnetic field coil.

From Fig. 14b, the stress of the eddy current coil in the three-coil mixed force forming scheme is less than that of the dual-coil attractive forming scheme, and the gap is between 3 and 5%. In the two schemes, although the short pulse width circuit parameters are the same, the current in the eddy current coil is reduced due to the series operation of the compensation coil and the eddy current coil in the three-coil mixed force scheme. Therefore, it can significantly improve the stress environment of the eddy current coil.

### Compensation coil

**Figure 15:**
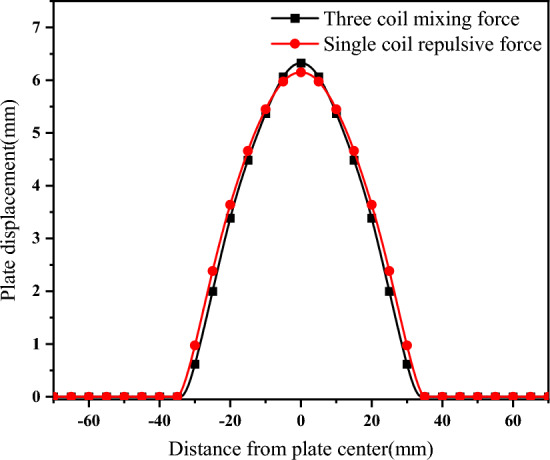
Single compensation coil repulsive force shaping at7.73kV.

The introduction of a compensation coil can improve the stress environment of the background magnetic field coil and eddy current coil. Its own stress cannot be ignored. When the compensation coil acts alone, it is equivalent to a single-coil repulsion forming scheme. As shown in Fig. 15, when the short pulse width voltage U_S_ is increased to 7.73 kV, the displacement of the sheet formed by the single compensation coil repulsion forming is basically similar to that of the three-coil mixed force forming.

**Figure 16:**
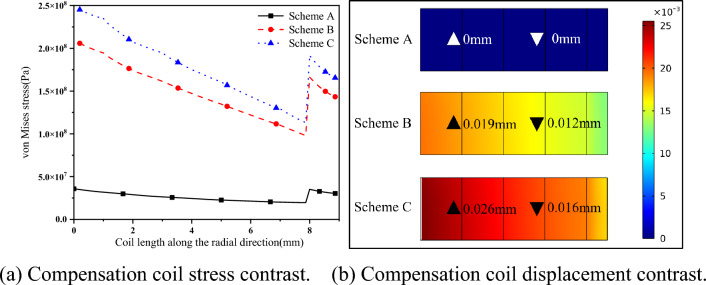
Comparison of stress and displacement of compensation coil.

As shown in Fig. 16, this section will compare the stress of the three-coil mixed force forming (Scheme A), the short pulse width power supply directly connected to the compensation coil forming (Scheme B) and the short pulse width voltage (U_S_ = 7.73 kV) single-coil repulsion forming (Scheme C) when the short pulse width current is maximum.

It can be seen from Fig. 16a that the stress of the compensation coil in Scheme A is one order of magnitude lower than that of Scheme B and Scheme C. The compensation coil of Scheme A is connected in series with the eddy current coil, and the number of turns of the eddy current coil is much larger than that of the compensation coil, so the resistance of the eddy current coil is much larger than that of the compensation coil. The current of the compensation coil in Scheme A is much smaller than that of Scheme B and Scheme C, and the magnetic field strength of the coil is reduced. So, the coil stress can be greatly improved.

From Fig. 16b, in Scheme A, the coil did not deform. In Scheme B and Scheme C, the coil produced obvious deformation, the deformation was 0.019 mm and 0.026 mm. The coil cannot be used safely, and there is a risk of rupture. Therefore, Scheme A can greatly optimize the stress environment of the compensation coil, reduce the risk of coil rupture and improve the service life of the coil.

## Conclusion

Aiming at the problem of magnetic field competition and eddy current competition in the sheet electromagnetic forming system of dual-coil attraction, a three-coil dual-power sheet electromagnetic forming system is proposed. The key is to introduce a compensation coil.

1. Compared with the dual-coil attractive forming, the magnetic field in the three-coil mixed force scheme is increased by 178%, and the eddy current is increased by 91%. It can be seen that the compensation coil can effectively improve the magnetic field competition and eddy current competition, thereby improving the electromagnetic force and achieving a better forming effect.
2. Under the same power supply parameters, the maximum displacement of the single-coil repulsion forming is 5.22 mm, the dual-coil attraction forming is 1.01 mm, and the three-coil mixed force forming is 6.89 mm. The three-coil mixed force forming scheme improves the energy utilization rate of the system.
3. The larger the number of turns of the compensation coil, the better the forming effect of the sheet in the three-coil mixed force scheme. When the compensation coil is 3 turns or less, the sheet forming effect is poor and there is still a competitive failure area. When the number of turns is greater than 3, the competition failure area disappears, and the displacement is increasing.
4. The three-coil mixed force forming can optimize the coil stress environment. During the forming process, the eddy current coil can achieve voltage division with the compensation coil and can achieve a better forming effect under smaller power supply parameters, further optimize the coil stress environment, and reduce the risk of coil rupture.

## Data Availability

All material data in this article are from previous work, the data is very reliable. All data generated or analysed during this study are included in this published article.
